# Medical Officers and the Supply of Drugs

**Published:** 1917-09-22

**Authors:** 


### MEDICAL OFFICERS AND THE SUPPLY OF DRUGS.

### The Practice in London Unions.

Having had applications from district medical officers for additional remuneration on account of the increased cost of drugs, Mr. W. R. Owen, the Deputy-Clerk, was asked by the Lewisham Board of Guardians to make inquiries as to the method adopted by other London Boards of Guardians for supplying drugs to their outdoor sick poor. He is now able to report replies from twenty-two Unions, which show that the Lewisham district officers are the only doctors in the Metropolis having to provide a surgery and to supply drugs at their own expense. Salaries vary considerably throughout the Metropolis, and are governed to a large extent by the number of orders received by the medical officer. For instance, the average in Lewisham, where drugs are supplied by the doctors, is 2*s.* 6*d.* per order; in Greenwich, 3*s.* 10*d.* per order; and in Woolwich 3*s.* 9*d.* per order. Subjoined is the average payment per order made to the district medical officers of the twenty-two Unions: Camberwell, 2*s.*; Chelsea, 5*s.* 3*d.*; Fulham, 2*s.* 1*d.*; Greenwich, 3*s.* 10*d.*; Hammersmith, 2*s.* 10*d.*; Hampstead, 6*s.*; Holborn, 8*s.* 4*d.*; Islington, 2*s.* 5*d.*; Lambeth, 2*s.* 10*d.*; Lewisham, 2*s.* 6*d.*; City of London, 15*s.* 2*d.*; Paddington, 3*s.* 3*d.*; Poplar, 2*s.* 5*d.*; Shoreditch, 2*s.* 6*d.*; Southwark, 4*s.* 6*d.*; St. George-in-the-East, 3*s.* 2*d.*; St. Marylebone, 3*s.* 11*d.*; Stepney, 3*s.*; Wandsworth, 2*s.* 11*d.*; City of Westminster, 6*s.* 8*d.*; Woolwich, 3*s.* 9*d.* The Bermondsey and Bethnal Green Guardians have adopted the modern method of putting the medical relief under the direction of their Infirmary Medical Superintendents, and the Lambeth Guardians have this system in force in four of their medical relief districts. At a recent interview between representatives of the district medical officers and the Lewisham Guardians it was stated that, in fixing the remuneration, the Guardians had in mind the extra medical fees received; but it was shown that these fees had considerably decreased of recent years, due, it was thought, to the effect of the National Insurance Act.

Mr. Owen adds that the whole question of the outdoor medical relief arrangements is one which might with advantage be thoroughly investigated, but having regard to the unsettled conditions now prevailing he thinks the Guardians would prefer to allow the matter to remain as at present until after the war.

We hope, however, that something will be done to increase the payments to the district medical officers of Lewisham, who provide both the surgery and the drugs.

